# Editorial: Aerospace health and safety: today and the future, volume II

**DOI:** 10.3389/fpubh.2025.1666680

**Published:** 2025-10-09

**Authors:** Mardi Crane-Godreau, Eileen McNeely, Philip Parks, Russell Tontz, Christopher Scheibler

**Affiliations:** 1 Health Education Outreach, Independent Researcher, Arlington, VA, United States; 2 Institute of Quantitative Social Science, Harvard University, Cambridge, MA, United States; 3 H21, Wayland, MA, United States; 4 Harvard Medical School, Boston, MA, United States; 5 School of Public Health, Harvard University, Boston, MA, United States

**Keywords:** aviation and aerospace, health and safety, fatigue, burnout, gravitation, balance, physiology, depression

**Figure 1:**
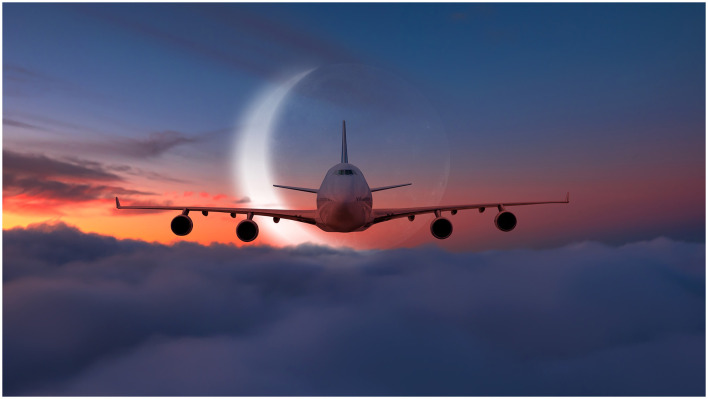
Aerospace health and safety: today and the future. Reproduced from “Airplane flying over tropical sea with crescent moon at amazing sunset” by muratart, licensed under Enhanced Image License.

Aviation is a cornerstone of modern commerce, defense, science, and leisure. In 2024 alone, the world's airlines carried an estimated 5 billion passengers, demonstrating their global importance. Now, we stand at the precipice of a new era. With the rise of commercial civilian spaceflights by companies such as Blue Origin, Virgin Galactic, and SpaceX, the dream of space travel is becoming a reality for non-professional astronauts. However, this expansion into a domain where environmental conditions differ widely from our terrestrial experience presents unprecedented challenges and questions regarding human health and safety (Figure 1).

In this second volume of *Aerospace Health and Safety: Today and the Future*, 12 new publications address complex health and safety concerns for personnel working in the air and in space. These studies can be broadly understood through the key themes of physiological adaptation, mental resilience, occupational health, and advanced diagnostics.

One central theme is the physiological challenge of adapting the human body to extreme gravitational changes. In *Peripheral skin cooling during gravitational challenges in parabolic flight*, Bothe et al. investigated a proof-of-concept experimental model that tested peripheral cooling (PC) as a countermeasure to cardiovascular instability during gravitational shifts. Their preliminary testing shows that heart rate and blood pressure fluctuations were reduced, leading them to advocate for further controlled studies to assess PC as a non-invasive countermeasure.

Optimizing balance and sensorimotor function is equally critical. Two articles in this volume report on adapting training for this purpose. In *The ground reaction force pattern during walking under vestibular-demanding task*, Wang Z. et al. examined changes in ground reaction force (GRF) during normal walking and under sensory-deprived conditions. They concluded that these methods could be used not only to detect changes pre- and post-mission but also to develop specific sensorimotor training programs “aimed at enhancing astronauts' abilities to navigate unpredictable sensory-conflicted conditions”. Recognizing that anatomical differences may influence outcomes, Zhang et al. explored sex differences. In *The sex effect on balance control while standing on vestibular-demanding tasks*, they reported that since both men and women are included in space programs, “it is essential to clarify how women differ from men when it comes to balance control.” Their work provides a fundamental reference for studying the vestibular system and designing tailored rehabilitation programs for male and female astronauts.

Beyond physical adaptation, maintaining mental health and peak performance under stress is paramount. In *The effect of Ashtanga-Vinyasa Yoga method on air force pilots' operational performance*, Santos et al. addressed the importance of “optimizing performance and bolstering physical health and mental resilience” in military pilots. Their manuscript describes a feasibility study of a 12-week yoga program during pilot training. If the program proves effective, the authors hypothesize “that this method will enhance operational performance and, subsequently, elevate flight safety”.

Burnout and stress pose significant threats to aviation safety. In *The mechanisms linking perceived stress to pilots' safety attitudes*, Yanzeng et al. studied the links between stress, cognitive flexibility, and burnout. Their results demonstrate a significantly negative correlation between pilots' perceived stress and their safety attitude, highlighting the critical role of cognitive flexibility and the complex impact of job burnout. The COVID-19 pandemic exacerbated these stressors. In *Challenges and support needs in psychological and physical health among pilots: a qualitative study*, Xu et al. reported that during the pandemic, the “health of pilots was not taken seriously.” Their study aimed to clarify these challenges to inform the development of a more scientific and comprehensive health system for civil aviation pilots.

Fatigue and sleep deprivation remain persistent operational risks. In *Comparison of effects of modafinil and caffeine on fatigue-vulnerable and fatigue-resistant aircrew*, Wingelaar-Jagt et al. described the potential benefits of stimulants during periods of sleep deprivation. The study confirmed “different degrees of performance degradation” and suggested that “stimulants might be especially useful for fatigue-vulnerable individuals.” Compounding the issue of fatigue, Shi et al. investigated sleep disorders in Chinese airline pilots. In *Association of age and night flight duration with sleep disorders among Chinese airline pilots*, they reported that aging and monthly night flight duration had a synergistic, negative effect on sleep, calling for urgent exploration and intervention. Finally, addressing trauma is essential for long-term crew health, and, In *Assessment policy of post-traumatic stress disorder in aviation*, Vuorio et al. suggested the global adoption of the International Classification of Diseases (ICD-11) criteria for stress disorders to standardize the assessment and treatment of aircrew.

To support these efforts, accurate screening and diagnostic tools are indispensable. In *Echocardiography screening of German military pilot applicants*, Guettler and Sammito analyzed 14.5 years of echocardiograms to determine how often cardiac diseases were diagnosed and influenced aeromedical decision-making, providing valuable data for screening protocols in high-hazard occupations. Similarly, understanding occupational risks is crucial. In *Prevalence and risk factors of occupational neck pain in Chinese male fighter pilots*, Yang et al. pointed to the need for “appropriate training schedules and a more holistic perspective on musculoskeletal protection” to mitigate a common ailment among fighter pilots. Even environments on Earth can offer insights. In *Short-term changes in chest CT images among individuals at low altitude after entering high-altitude environments*, Wang P. et al. reported on pathologies such as spontaneous mediastinal emphysema that occur during high-altitude adaptation, offering a terrestrial analog for understanding physiological stress.

This Research Topic of studies adds crucial links for safeguarding human capital in the rapidly evolving aerospace sector. The combined insights into physiological countermeasures, mental resilience, and occupational health create a clear, evidence-based agenda. This work supports the ongoing need to manage risk and enhance performance, ensuring that the next great leaps in aviation and space exploration are both ambitious and safe.

