# Correction: Editorial: Aerospace health and safety: today and the future, volume II

**DOI:** 10.3389/fpubh.2025.1764144

**Published:** 2026-01-29

**Authors:** Frontiers Production Office

**Affiliations:** Frontiers Media SA, Lausanne, Switzerland

**Keywords:** aviation and aerospace, health and safety, fatigue, burnout, gravitation, balance, physiology, depression

There was a mistake in the above article as published whereby Figure 1 was not published. Figure 1 and accompanying figure caption appear below.

**Figure 1:**
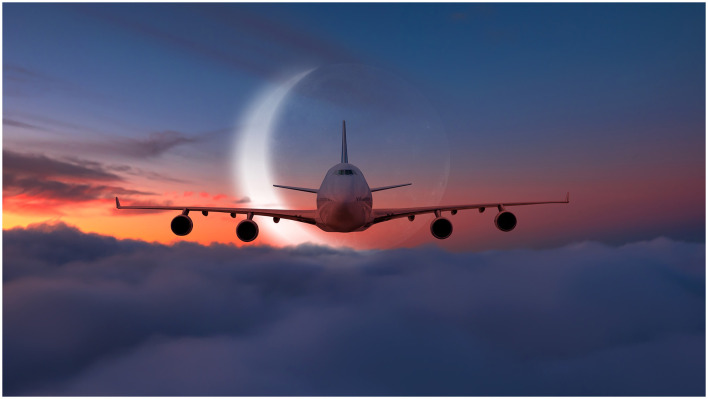
Aerospace health and safety: today and the future. Reproduced from “Airplane flying over tropical sea with crescent moon at amazing sunset” by muratart, licensed under Enhanced Image License.

The original version of this article has been updated.

